# Partial recovery of voiding function in female mice following repeated psychological stress exposure

**DOI:** 10.1371/journal.pone.0266458

**Published:** 2022-04-21

**Authors:** Eliza G. West, Catherine McDermott, Russ Chess-Williams, Donna J. Sellers

**Affiliations:** Faculty of Health Sciences and Medicine, Centre for Urology Research, Bond University, Gold Coast, Australia; University Medical Center Utrecht, NETHERLANDS

## Abstract

Psychological stress causes bladder dysfunction in humans and in rodent models, with increased urinary frequency and altered contractile responses evident following repeated environmental stress exposure. However, whether these changes persist after removal of the stressor is unknown, and the aim of this study was to determine if stress-induced changes in voiding behaviour and bladder function recover following removal of the stressor. Adult female mice were allocated to three groups: Unstressed, Stressed or Stressed + Recovery. Animals in the stressed groups were exposed to water avoidance stress for 1h/day for 10-days, with unstressed animals age-matched and housed under normal conditions. For recovery studies, animals were housed without stress exposure for an additional 10-days. Voiding behaviour was assessed periodically and animals sacrificed on day 10 (Unstressed and Stressed) or day 20 (Unstressed and Stressed + Recovery). Isolated whole bladder studies were used to assess compliance, urothelial mediator release and contractile responses. Exposure to stress increased plasma corticosterone levels almost three-fold (P<0.05) but this returned to baseline during the recovery period. Contractile responses of the bladder to carbachol and KCl were also increased following stress, and again fully recovered after a 10-day stress-free period. In contrast, stress increased urinary frequency four-fold (P<0.001), but this did not return fully to baseline during the recovery period. Bladder compliance was unchanged by stress; however, it was increased in the stressed + recovery group (P<0.05). Thus, following a stress-free period there is partial recovery of voiding behaviour, with an increase in bladder compliance possibly contributing to the compensatory mechanisms.

## Introduction

Numerous clinical studies have reported a correlation between psychological stress and bladder dysfunction, with stress linked to development of bladder symptoms or worsening symptom severity. This is supported by evidence from experimental studies using various rodent models of psychological stress which have shown that stress plays a causal role in the development of bladder dysfunction. Our research group and others has shown that an overactive voiding phenotype develops in female mice and rats following water avoidance stress, with increased urinary frequency evident in voiding behaviour studies [1–3]. This contrasts with the urinary retention seen in male mice due to repeated bouts of social defeat but not witness trauma or restraint stress [4,5] and male rats following water avoidance stress [6]. These changes in voiding have been linked to local inflammation, bladder wall hypertrophy, alter detrusor contractile responses, in addition to changes in central control mechanisms [2,7–9].

Few studies have assessed recovery of physiological functions altered by psychological stress. A study that investigated the effects of water avoidance stress on visceral and somatic nociception, colonic motility, and anxiety-like behaviour reported that chronic stress in rats resulted in persistent visceral hyperalgesia lasting at least 1 month [10]. Thirty-days of social defeat stress in male mice was used to stimulate psychoemotional stress led to anxious and depressive symptoms. They found that even after cessation of psychological stress, the male mice continued to show symptoms of anxiety and depression in behavioural tests over 2-week study period [11]. Similarly, urinary retention in male mice caused by 4-weeks of social defeat stress, persisted after 4-weeks stress-free recovery with no benefit to use of the anxiolytic fluoxetine observed [12]. Clinical examination of cortisol responses to psychological stress found that non-depressed and clinically depressed individuals exhibited similar baseline cortisol levels. In the recovery period however, clinically depressed patients had a significantly higher cortisol level compared to their non-depressed counterparts [13]. Several clinical studies have also observed that delayed recovery from psychological stress leads to poor cardiovascular outcomes and may predict earlier death [14]. The only study to our knowledge that reported the persistence of bladder dysfunction caused by water avoidance stress stated that micturition frequency increased with water avoidance stress and that these voiding changes persisted for 1 month. However, no data was presented in the paper to support these persistent changes [1].

The potential for recovery of bladder function following cessation of water avoidance stress exposure has not been clearly investigated or reported. Given the intermittent and recurring nature of psychological stress it is an important aspect that must be considered when assessing related physiological changes. Therefore, the aim of this study was to determine if the voiding dysfunction and altered bladder mechanisms induced by water avoidance stress persists following 10-days stress-free recovery.

## Methods

### Water avoidance stress model

All procedures were performed in accordance with the Australian Code for the Care and Use of Animals for Scientific Purposes and with the approval of the University of Queensland Animal Ethics Committee. Adult female C57/Bl/6J mice (12–14 weeks in age; n = 6 in each group) housed in pairs under environmentally controlled conditions, with 12-hour light-dark cycles, with access to food and water ab libitum. Animals were randomly allocated to into the following experimental groups: Unstressed, Stressed or Stressed + Recovery.

Water avoidance stress was performed as previously described [2,3]. Briefly, mice in both the Stressed and Stressed + Recovery groups were placed individually on a pedestal surrounded by water for 1 hour/day for 10 consecutive days (typically started at 11am immediately following voiding analysis). After each 1-hour stress exposure, mice were returned to their normal housing. The unstressed group consisted of age-matched control mice housed under normal conditions and were not exposed to water avoidance stress protocols. Following the 10-day stress protocol mice in the Stressed + Recovery group were housed under normal conditions without stress exposure for an additional 10-days.

### Voiding Pattern Analysis (VPA)

Changes in voiding behaviour were assessed as previously described to determine how water avoidance stress and stress-free recovery affects urinary frequency, total voided volume, average void size and number of small voids, compared to unstressed animals [2]. VPA was performed prior to (baseline) and at intervals (1, 3, 5, 7 and 10 days) during the WAS protocol in all animals. VPA was also conducted on days 11, 13, 15, 17 and 20 (for Unstressed and Stress + Recovery animals) during which time all animals were housed under normal conditions without stress exposure. Mice were place individually for 4 hours, at the beginning of the light cycle in cages lined with hardened ashless filter paper, (Filtech; Quantitative 2um grade 225). Animals had access to food and drinking water during this time. Filter papers were collected, and urine spots detected using a Molecular Imager ChemiDoc XRS ultraviolet transilluminator (BioRad, California USA). The papers were photographed, digitized, and then analysed using Image J software.

### Isolated whole bladder preparation

Functional bladder assessments were carried out using an isolate whole bladder preparation and set up as previously described [4,15]. Animals in the Stressed group were sacrificed by cervical dislocation 24 hours following the final water avoidance stress exposure, or on day-20 for Unstressed and Stressed + Recovery groups. At the time of sacrifice, a venous blood sample was taken and plasma corticosterone levels quantified using the Corticosterone Competitive ELISA (Invitrogen) according to the manufacturer’s instructions. Blood samples were collected in the morning to avoid circadian variation in corticosterone levels.

The urinary bladder was isolated, and a three-way catheter was inserted through the urethra. The urethra and ureters were ligated, and the bladder was placed into a bath of gassed (95%O_2_/5% CO_2_) Krebs-bicarbonate solution (composition in mM: NaCl 118, NaHCO_3_ 24.9, CaCl_2_ 1.9, MgSO_4_ 1.15, KCl 4.7, KH_2_PO_4_ 1.15, and D-glucose 11.7) at 37°C. The three-way catheter was attached to an infusion pump to allow bladder filling, a pressure transducer to record intravesical pressure, and an outflow syringe to collect intraluminal fluid and allow bladder emptying. Intravesical pressure was measured using a pressure transducer (GlobalTown Microtech, Sarasota, FL) connected to a PC via a PowerLab data acquisition system (AD Instruments, Sydney, Australia), using LabChart 7 software (AD Instruments). Bladder distensions were performed by intravesical infusion of saline at a rate of 30 μL/min up to a maximum pressure of 40 mmHg to assess viability, and to 20 mmHg for all further distensions.

Intraluminal fluid was collected via the catheter following distension to 20 mmHg, in addition to a sample of serosal fluid, to determine if changes in release of urothelial signalling mediators occurs. Samples were stored at -80°C until analysis of ATP and acetylcholine (ACh) levels. Quantification of ATP and ACh was carried out using the ATP Determination Kit (Molecular Probes), and the Acetylcholine Amplex Red Assay Kit (Molecular Probes) respectively. The assays were performed according to manufacturer instructions, with luminescence and fluorescence (excitation 540, emission 590 nm) measured, using a Modulus micro-plate reader (Promega).

Following bladder distension to 20 mmHg, bladders were allowed to equilibrate/accommodate for approximately 60 minutes, during which time spontaneous phasic activity was measured as (i) the frequency of spontaneous contractions per minute and (ii) the amplitude measured as the change in intravesical pressure from the trough to peak of the contractions.

Changes in nerve-mediated contractile bladder responses were assessed by electric field stimulation (EFS). The bladder was electrically stimulated (0.1ms pulse-width, 50 V) for 5 seconds, every 100 seconds at 1–20 Hz. Bladders were stimulated at each frequency until 3 consistent responses were obtained and contractions were measured as the increase in intravesical pressure from baseline. EFS was repeated at 20 Hz in the absence and presence of atropine (1 μM) to block muscarinic receptors and αβ-methylene ATP (10 μM) to desensitize P2X receptors and thus remove cholinergic and purinergic components, respectively. Neurogenic origins of the pressure responses were confirmed by addition of tetrodotoxin (0.1 μM), which abolished responses to EFS.

Intravesical pressure responses to pharmacological agents were also assessed by addition of cumulative concentrations of the muscarinic agonist carbachol, and relaxations to the β-adrenoceptor agonist isoprenaline following precontraction with carbachol (1 μM). Non-receptor mediated contractile bladder responses were also assessed using KCl (60 mM). All contraction and relaxation responses were measured as change in pressure from baseline.

### Data and statistical analysis

All experiments were randomized, with 6 mice per experimental group and each experimental protocol started on a different day. Results are expressed as mean ± standard error of the mean (SEM). Data were analysed using ordinary one-way ANOVA with Dunnett multiple comparisons test or repeated measures two-way ANOVA with Bonferroni’s multiple comparisons test, using GraphPad Prism version 6 software (GraphPad, San Diego, CA). Significance levels were defined as P<0.05 (*).

## Results

### Bladder weight, water consumption and voiding behaviour

**Fig 1:**
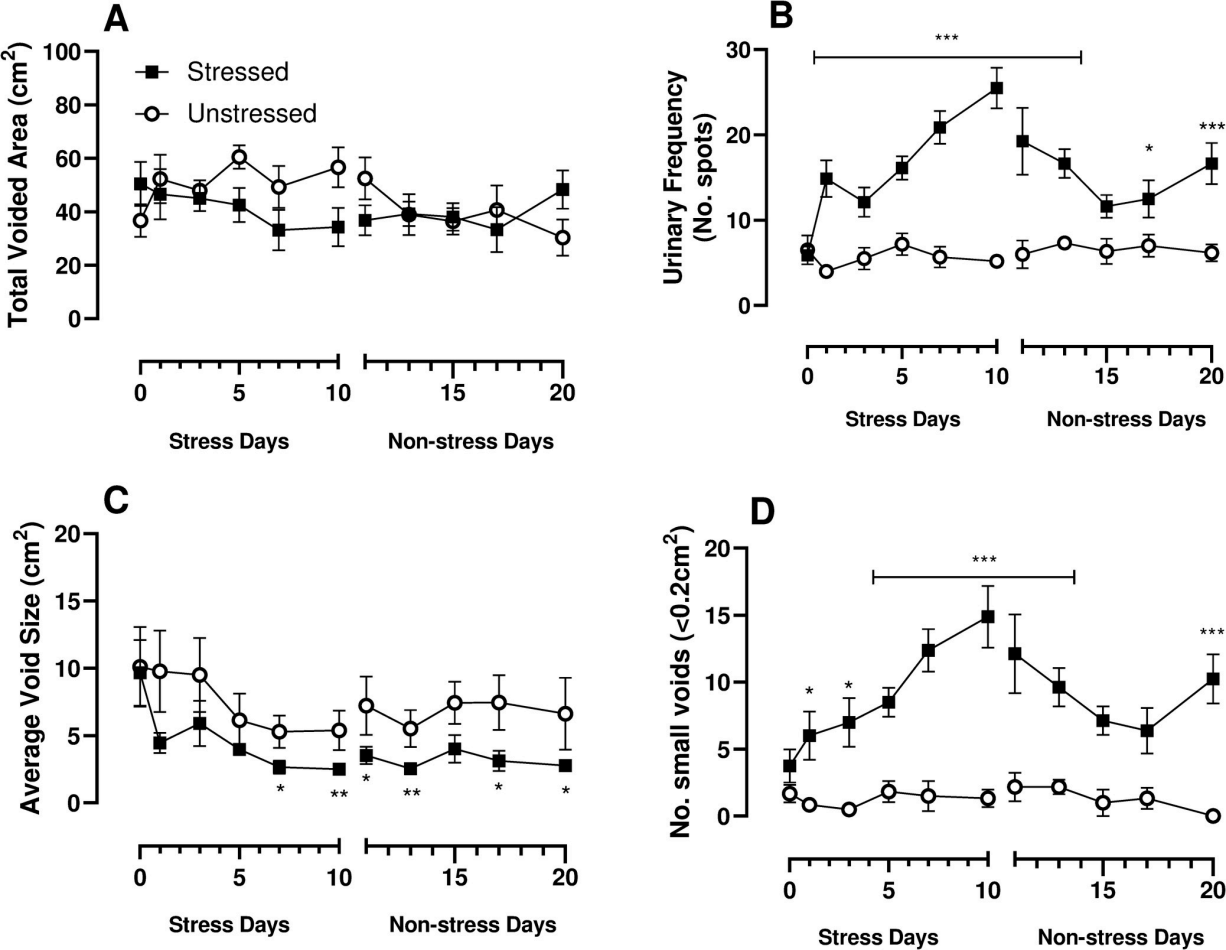
Changes in voiding behaviour measured in mice during 10-day stress exposure period and over the following non-stress days compared to unstressed controls. Voiding was assessed in terms of change in A) total voided area, B) urinary frequency, C) average void size and D) number of small voids. Data represents mean ± SEM (n = 6) and was analysed using two-way ANOVA with Bonferroni’s multiple comparisons test (*p<0.05, **p<0.01 and ***p<0.001 vs unstressed).

No significant difference in bladder weight, body weight or water consumption as detected between the experimental groups (Table 1). Voiding pattern analysis was used to assess stress induced changes in voiding behaviour and recovery of voiding function. Total voided area which represents urine output was unchanged over the 10-days of stress and was similarly unchanged during the stress-free recovery period indicating urine production was unchanged (Fig 1A). Voiding frequency increased in stressed mice over the initial 10-day stress period compared to the unstressed group. Over the following 10-days of stress-free recovery, the voiding frequency decreased although not to baseline, remaining significantly elevated (p = <0.001) compared to the unstressed group on day 20 (Fig 1B). The average void size was significantly reduced by stress and remained so during the stress-free recovery days (Fig 1C). Similarly, the number of small voids was also increased over the 10-days of stress and was still increased in the following 10-day stress-free period (p = <0.001) (Fig 1D).

**Table 1:** Body weight, water consumption during VPA and bladder weight (at time of sacrifice) in Unstressed, Stressed and Stressed + Recovery mice.

	*Unstressed*	*Stressed*	*Stressed + Recovery*
***Body weight (g)***	21.2 ± 0.28	18.5 ± 0.67	20.6 ± 0.67
***Bladder weight (mg)***	23.1 ± 1.13	20.78 ± 0.53	22.3 ± 1.43
***Water consumption (g)***	0.95 ± 0.06	0.79 ± 0.23	1.15 ± 0.12

### Plasma corticosterone and isolated whole bladder responses

**Fig 2:**
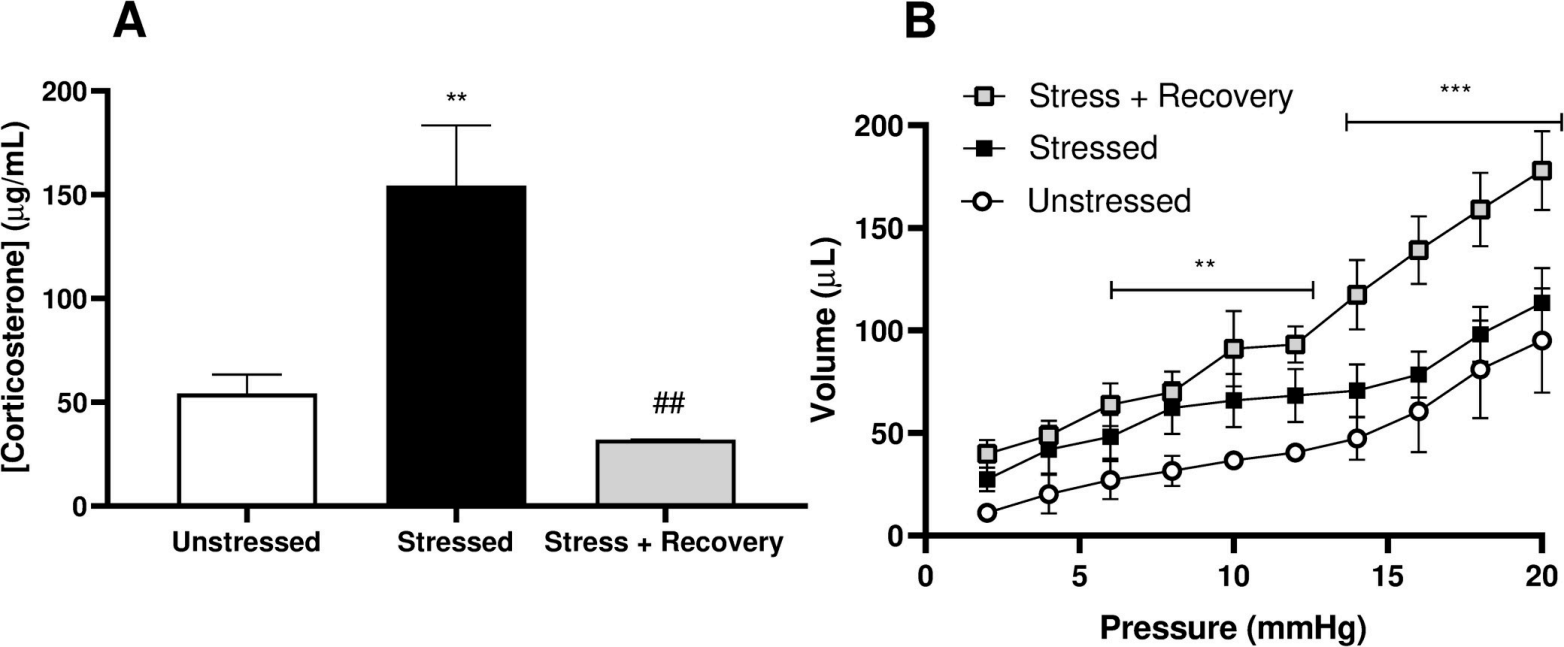
A) Plasma corticosterone measured in animals from unstressed, stressed and stress + recovery groups. B) Volume-pressure relationship in isolated whole bladders. Data represents mean ± SEM (n = 6) and was analysed using (A) one-way ANOVA with Tukey’s multiple comparisons test or (B) two-way ANOVA with Tukey’s multiple comparisons test (***p<0.01 and ***p<0.001 vs unstressed; ##p<0.01 vs stressed).

At the time of sacrifice a blood sample was taken for determination of the hormonal stress response. Plasma corticosterone levels were significantly increased in the Stressed group at day-10 (154.20 ± 29.24 μg/mL (p = 0.0048)) compared to the Unstressed group (54.14 ± 9.14 μg/mL), with corticosterone levels significantly decreased and returned to unstressed levels after 10-days of stress-free recovery (32.06 ± 0.10 μg/mL) (p = 0.0031) (Fig 2A).

The volume-pressure relationship of isolated whole bladders during distension was not significantly changed by stress with a 95 ± 10 μL saline stored in bladders from unstressed mice at an intravesical pressure of 40mmHg compared to 113 ± 17 μL in the stressed group. However, 10-days stress-free recovery resulted in a significant increase in bladder compliance with a larger volume of saline (178 ± 19 μL) stored at the same pressure (p = 0.006 vs Unstressed; p = 0.03 vs Stressed) (Fig 2B).

**Fig 3:**
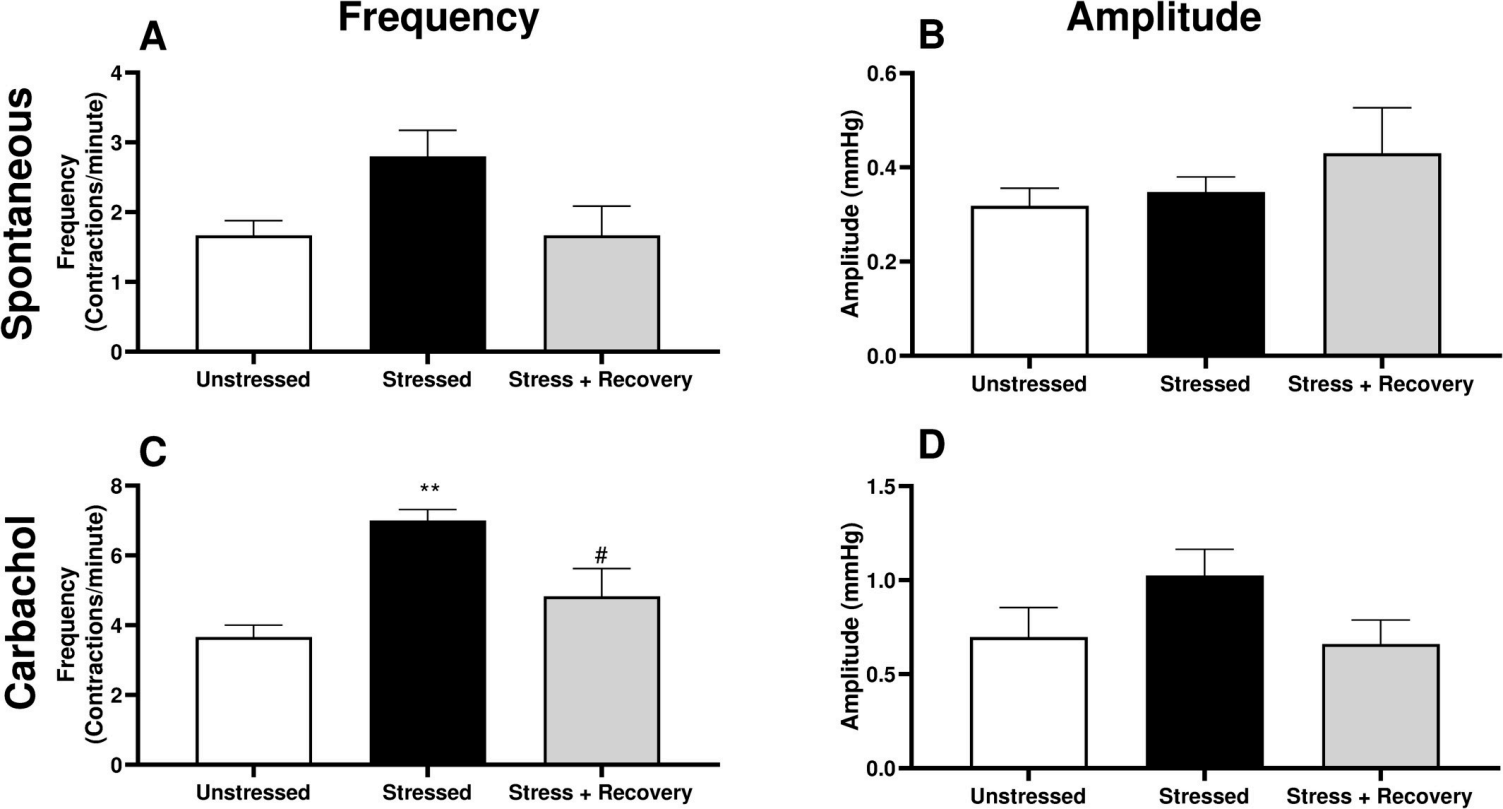
(A-B) Spontaneous and (C-D) carbachol (1μM) stimulated phasic contractions in isolated whole bladders from unstressed and stressed mice, and following 10-days stress free, measured as (A&C) frequency and (B&D) amplitude of phasic contractions. Data represents mean ± SEM (n = 6) and was analysed using one-way ANOVA with Tukey’s multiple comparisons test (**p<0.01 stressed vs unstressed; #p<0.05 stressed vs stress + recovery).

Spontaneous phasic activity was measured during accommodation following bladder distension to 20mmHg, with no significant change in the frequency or amplitude of spontaneous contractions observed (Fig 3A and 3B). However, the frequency, but not amplitude, of phasic contractions following stimulation with the muscarinic agonist carbachol was significantly increased in the Stressed group compared to the Unstressed group, with stress-free recovery significantly reducing this change (Fig 3C and 3D).

**Fig 4:**
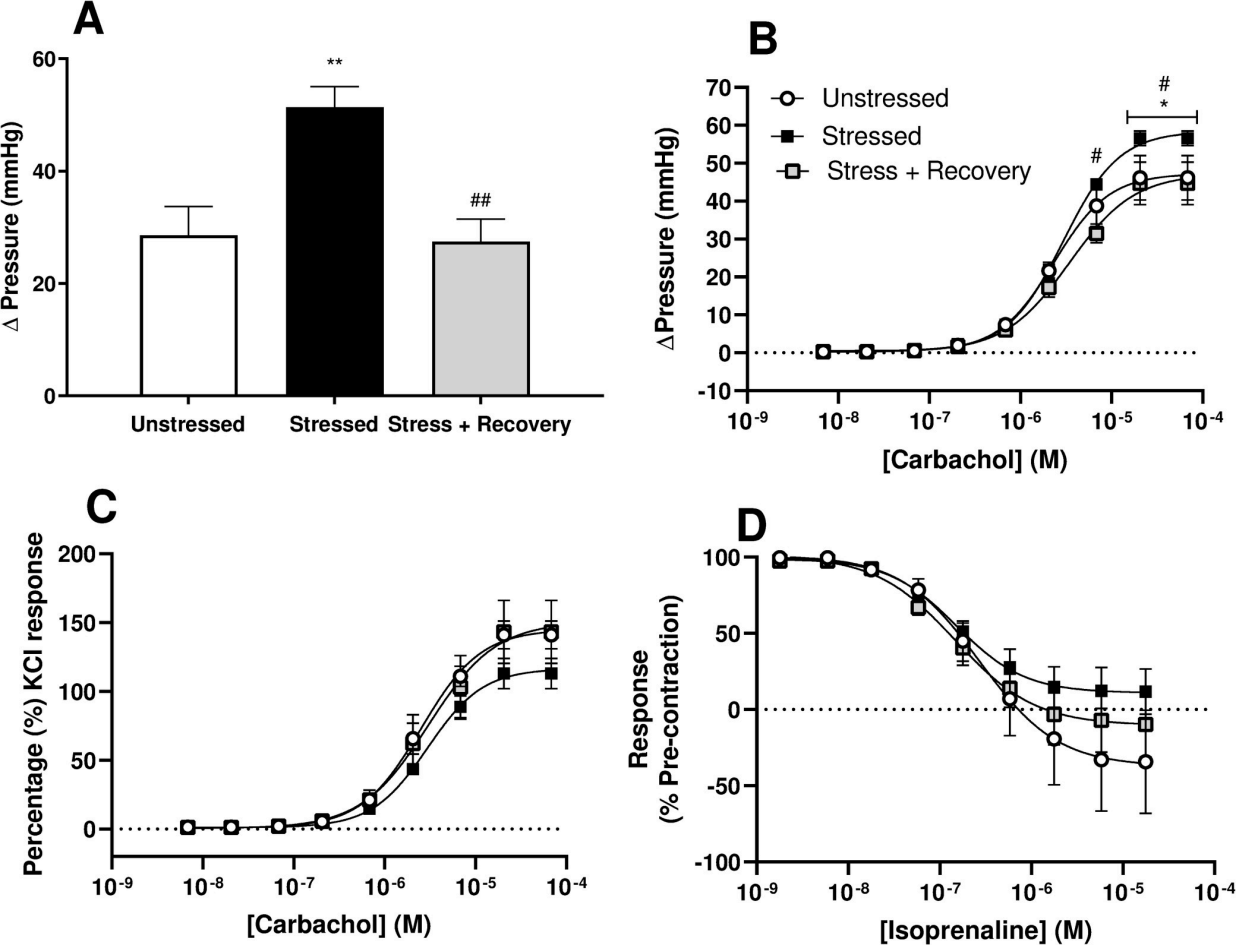
Responses stimulation with A) KCl, B&C) the muscarinic agonist carbachol, and D) beta-adrenoceptor agonist isoprenaline following precontraction to carbachol (1μM) in isolated whole bladders from unstressed, stressed and stress + recovery mice. Data represents mean ± SEM (n = 6) and was analysed using one-way ANOVA (A) or two-way ANOVA (B-D) with Tukey’s multiple comparisons test (* Stressed vs Unstressed; # Stressed vs Stressed + Recovery).

Receptor-independent contractions to KCl and maximal responses to muscarinic stimulation with carbachol in isolated whole bladders were significantly increased in the Stressed group compared to the Unstressed group, while 10-days stress-free recovery returned responses to unstressed levels (Fig 4A and 4B). When carbachol responses were normalised to the KCl response, no significant difference in maximal contractile responses was detected (Fig 4C). Bladder relaxation to the beta-adrenoceptor agonist isoprenaline was not significantly changed across the experimental groups (Fig 4D).

**Fig 5:**
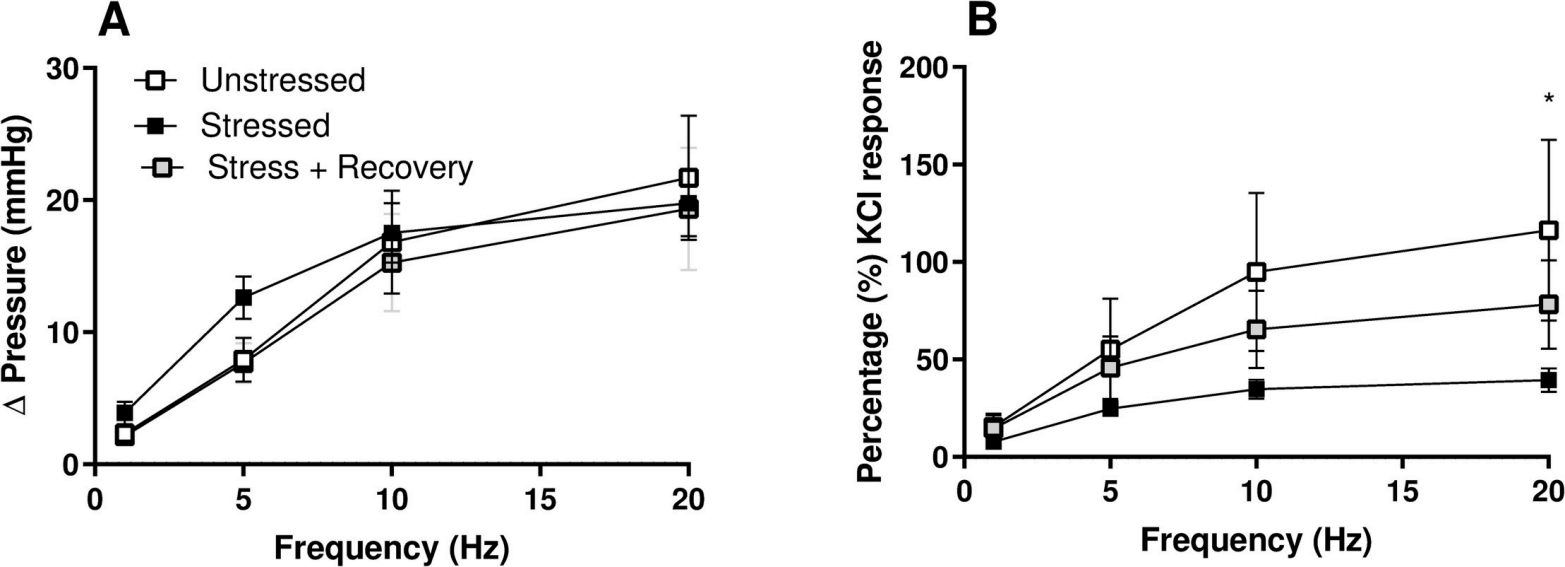
Nerve evoked contraction to electric field stimulation in isolated whole bladders from unstressed and stressed mice, and following 10-days stress-free recovery, presented as A) change in pressure from baseline and B) as a percentage of the KCl response. Data represents mean ± SEM (n = 6) and was analysed using two-way ANOVA (*Unstressed vs Stressed).

Stress did not alter nerve-mediated bladder contractions and the responses to EFS were also not changed in stressed mice following the 10-days stress-free period (Fig 5A). When the EFS responses were normalised to the KCl response, nerve mediated contractions in the stress group were depressed compared the unstressed group, this difference being significant at 20 Hz (Fig 5B). The addition of atropine to block cholinergic bladder responses, decreased the pressure response to EFS at 20Hz by 21.6 ± 3.62% in the control group, with a similar change observed in the stressed (26.9 ± 4.9%) and recovery (16.9 ± 5.5%) groups. Desensitization of purinergic receptors with αβm-ATP reduced the response to EFS by a further 54.9 ± 4.2% in bladders from unstressed animals, again with no significant difference between the groups (65.6 ± 4.5% Stressed; 49.8 ± 6.2% Stressed + Recovery).

**Fig 6:**
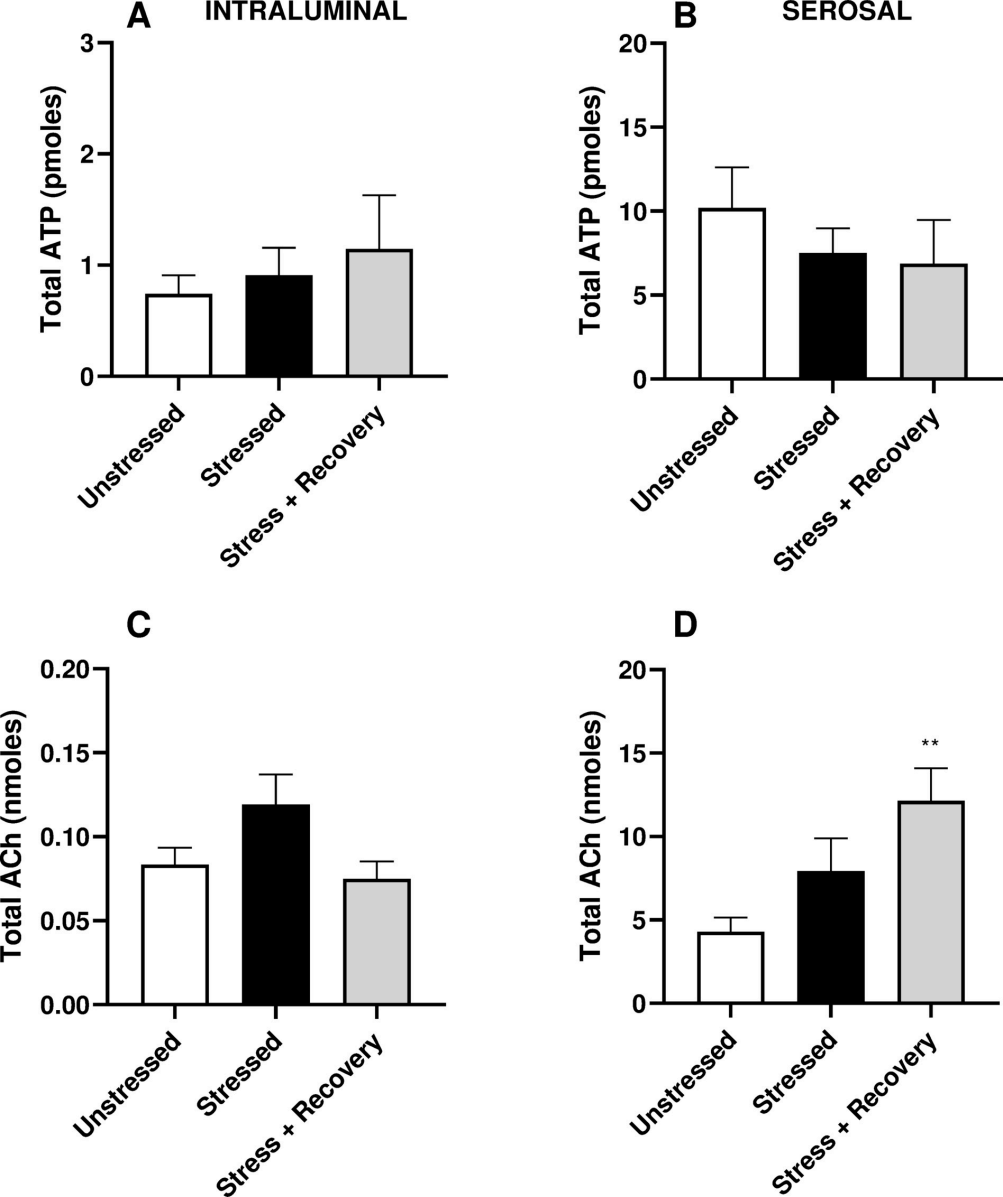
Effects of stress and 10-days stress-free recovery on total release of (A-B) ATP and (C-D) ACh from isolated whole bladders following distension to 20mmHg into the (A&C) intraluminal and (B&D) serosal fluid compared to unstressed controls. Data represents mean ± SEM (n = 6) and was analysed using one-way ANOVA with Tukey’s multiple comparisons test (**p<0.05 vs Unstressed).

Serosal and intraluminal ATP levels were unchanged across all experimental groups (Fig 6A and 6B). Similarly, intraluminal ACh levels were not significantly altered by 10-days exposure to WAS, nor did 10-days stress-free recovery affect release of this signalling mediator (Fig 6C). However, release of ACh into the serosal fluid was significantly increased in the Stressed + Recovery group compared to the unstressed group (p = 0.007) (Fig 6D).

## Discussion

The aim of this study was to determine if there is recovery from the voiding dysfunction observed in the female water avoidance stress model. Much of the previous research has focused on the immediate impact of chronic psychological stress on behaviour and physiological function. There has been some more recent work investigating recovery post-stress on other organ systems, with evidence that stress-induced hippocampal structural and functional changes do not simply return to the original unstressed state following a recovery period, with significant changes in hippocampal gene expression evident in post-stress recovery groups [16,17]. Chronic psychological stress results in an irreversible decrease in anti-oxidant defences in the fallopian tubes of rats measured after a 4-week recovery following exposure to restraint stress for 4, 8 or 12 weeks, unlike uterine changes which returned to unstressed control levels following 4 and 8 weeks, but not 12 week stress exposure [18]. Chronic social defeat stress results in urinary retention that persists for at least 4-weeks following cessation of stress in young male mice, accompanied by persistent elevation in CRF expression in Barrington’s nucleus and an increase in bladder mass [12].

We are aware of only one experimental study that reported on the persistence of voiding dysfunction following chronic water stress exposure, which claimed that changes in micturition lasted for approximately 1 month in female rats following water avoidance stress, with a mean duration of 24 days, although surprisingly supporting data was not presented in the paper [1]. As reported previously, water avoidance stress results in an overactive voiding phenotype in mice, with voiding increased over the 10-days stress exposure. While voiding frequency did decrease over the following 10-days recovery period, voiding frequency remained significantly elevated compared to unstressed controls, with an apparent spike at day 20. The same trend was also observed in the number of small voids (<0.2cm^2^) and average void size was consistently reduced. This demonstrates the long-term effect of psychological stress on bladder function.

Corticosterone levels have been documented to increase in rodent stress models [19,20]. Here we demonstrate that corticosterone levels decreased in the stressed mice after 10-days of stress-free recovery, returning to unstressed control levels. This indicates that the hormonal response to psychological stress recovers, but this is not associated with full recovery of bladder function.

Changes in bladder compliance manifest with long term changes in bladder morphology. The extracellular matrix of the bladder is made up of Type I and Type III collagen, which help to maintain tension and smooth muscle binding, leading to decreased contractility and compliance [21] and increased bladder elasticity and contractility of smooth muscle respectively [22]. One study has found that after chronic stress of female mice, both type I and III collagen were increased in bladders, which demonstrates that stress can induce pathological changes in bladder stability [23], and while no change in bladder compliance was observed in the stressed group in this study, compliance was increased following a period of recovery after stress. It suggests that changes in the collagen smooth muscle ratio occurs as a compensatory mechanism in the recovery phase post-stress, thereby increasing compliance in the recovered bladders. It is interesting that voiding behaviour had not fully recovered given the increase in compliance observed. This may be explained by bladder hypersensitivity caused by stress [24,25]. We have recently shown that water avoidance stress results in increased bladder afferent activity in both low and high threshold fibres, specifically at low bladder pressures within the physiological range [25]. This afferent hypersensitivity may be persistent and therefore contributing to continued voiding dysfunction regardless of cessation of stress, causing voiding to occur at lower bladder pressures despite increased compliance. Also of note is that the volume/pressure relationship is similar between groups at lower pressures, at which micturition is likely to be triggered if bladder afferent activity remains increased.

Detrusor smooth muscle exhibits spontaneous phasic activity of myogenic origin [26,27]. This is reported to be dependent on calcium entry mechanisms and potassium channels and modulated by the presence of the urothelium [27,28]. In animal models of bladder dysfunction [29,30] and in patients with bladder overactivity, enhanced phasic activity has been linked to the pathophysiology of observed bladder changes [31]. The increased frequency of phasic contractions observed in the stress group may therefore contribute to the overactive voiding pattern seen with stress. Stress-free recovery reduced the frequency of contractions and may therefore contribute to the partial recovery of voiding behaviour observed in these female mice.

Nerve mediated bladder responses to EFS, at all frequencies, were not affected by stress or recovery despite a general increase in contractility seen with stress. However when normalised to the KCl response, nerve-evoked contractions were depressed in the stressed group as reported previously [3], but not in the recovery group. With no change observed in the relative contribution of ATP and ACh to neurotransmission in the bladder, these changes would suggest reduced neurotransmitter release with stress and recovery of these changes post-stress. Similarly, there was also recovery of the enhance contractile responses observed to receptor-dependent and independent bladder contractions.

Interestingly, serosal ACh was significantly increased in the recovery group. Studies of non-neuronal ACh have found that urothelial cells express high-affinity choline transporter 1 as well as choline acetyltransferase and carnitine acetyltransferase [32]. While vesicular release of ACh occurs in nerve terminals, non-vesicular release has been shown to occur from the urothelium, due to the absence of the vesicular acetylcholine transporter. The same study also identified organic cation transporters 1 and 3, which are important for the release of ACh [33]. The role of ACh is still being identified, however, there is some evidence that the neurotransmitter influences sensory nerve activity and stimulates the release of urothelial derived inhibitory factor (UDIF) [34]. It could be theorised that enhanced ACh release results in greater release of UDIF, that then acts to inhibit detrusor muscle contraction in the recovery group decreasing the enhanced contractility caused by stress [35]. There is conflicting evidence regarding the role of urothelial ACh in modulating sensory nerve activity, with Daly et al reporting that muscarinic pathways depress sensory transduction in the bladder, while others claim there is a stimulatory effect [36–38]. Due to the conflicting nature of this evidence, it is difficult to interpret how enhanced ACh release plays a role during post-stress recovery; potentially improving voiding behaviour by desensitizing bladder afferent or alternatively preventing full recovery by sensitizing sensory nerves. The persistent elevation in voiding frequency and decreased average void size following a stress-free period, despite increased bladder compliance and recovery of detrusor contractile responses, suggests that there may be sensitization of bladder afferent nerves and/or alterations to central micturition control centre contributing to the stress induced voiding dysfunction. Stress is known to result in pro-inflammatory effects by activating mast cells, with increased total and activated mast cells observed in bladder wall specimens [1,39], with their presence linked to bladder pathologies. Histamine along with other inflammatory mediators is released upon mast cell activation and has been reported to increase the sensitivity of bladder afferent nerves to distension [40], and affect the frequency of spontaneous contractions in the urothelium and lamina propria [41]. While it is unknown if mast cell infiltration persists following cessation of stress, previous research has shown that ACh plays a role in activation of mast cell degranulation via muscarinic receptors [42]. The elevated ACh release observed here following the recovery period may contribute to the continued voiding dysfunction caused by stress via release of histamine.

The results presented here indicate that psychological stress affects bladder function, evident in the increased voiding frequency and increased contractile responses. There was a non-specific increase in detrusor contraction, which appears to be offset by the efferent innervation/neurotransmission. A period of stress-free recovery appeared to reduce the increased contractility induced by stress and was associated with enhanced bladder compliance. Interestingly, while voiding frequency was reduced following stress-free recovery, it remained elevated compared to unstressed controls which indicates another underlying mechanism may be responsible, possibly involving sensory nerves.

## Supporting information

S1 File (XLSX)
